# “Mindful eating: a comparative study between medical and non-medical students of Tanta University”

**DOI:** 10.1186/s41043-025-01232-3

**Published:** 2026-02-06

**Authors:** Asmaa Mohammad, Enas Kassim, Maha El-Sharawy, Ali Ali Elsherbini, Abdelaziz Farouk Eldeeb

**Affiliations:** Public Health and Community Medicine, Faculty of Medicine, Tanta University, Tanta, Egypt

**Keywords:** Mindfulness, Mindful eating, Mindless eating, Medical students, Eating behaviour

## Abstract

**Background:**

Mindful eating serves as a therapeutic intervention for recognizing and gradually altering the established daily eating habits and patterns.

**Objectives:**

This study aims to estimate the prevalence of adoption of principles of mindful eating among medical and non-medical university students and to detect the effect of adoption of mindful eating on the Body Mass Index of the studied subjects.

**Methods:**

A cross-sectional study was conducted among 576 undergraduate students using a structured self-administered questionnaire comprising sociodemographic data, perceived weight and height and Mindful Eating Questionnaire.

**Results:**

The majority of the studied students (94.4%) were used to adopt Mindful eating. The percentages of medical and non-medical students were used to adopt Mindful eating were (97.6% and 91.3%, respectively). A statistically significant association was detected between the type of the study (faculty) and adoption of mindful eating (P-value = 0.001). Medical study permits adopting mindful eating by about 73.8% more than non-medical studies. Medical male students showed higher scores for the disinhibition subscale than females (P-value = 0.025) while non-medical female students showed higher scores for the awareness subscale than males (P-value = 0.006). Nearly three fourths of the studied subjects (74.3%) had Body Mass Index ≥ 25. The percentage of medical students (17.4%) having Body Mass Index ≥ 25 was less than the percentage of their non-medical peers (34.0%). The association between the type of the study (faculty) and Body Mass Index categories was statistically significant (P-value = < 0.001). The higher scores on the Mindful Eating Questionnaire overall and on each of the categories except the awareness score had been associated with lower Body Mass Index. Most of the students adopting Mindful eating (75.4%) had Body Mass Index < 25 as compared to their peers who did not adopt Mindful eating where more than one third of them (43.8%) had Body Mass Index ≥ 25. The association between Mindful eating summary score categories and Body Mass Index categories was statistically significant (P-value = 0.016). A significant negative weak correlation was detected between Mindful eating summary score categories and Body Mass Index categories (P-value = 0.044).

**Conclusion:**

Academic background reinforces the potential of mindful eating as a viable strategy for promoting healthier eating habits and effective weight management among university students.

## Introduction

Mindfulness is a process-oriented, rather than an outcome-driven; behavior [1] concerned with doing anything consciously [2]. It is based on an individual’s experience of the moment [1] Mindful eating (MFE), a practice rooted in mindfulness, involves paying full attention to the experience of eating, recognizing hunger and satiety cues, and being aware of the physical and emotional aspects of food consumption [3], without judgment [2]. The individual focuses on appreciating the experience of food and is not concerned with restricting intake [1]. The goal is to promote a more enjoyable meal experience and understanding of the eating environment. MFE is also tied to food sustainability, with increasing awareness about reducing food waste, choosing local and seasonal foods, and supporting ethical food sourcing [4, 5]. 

To practice MFE, people should focus on their meals, shew slowly, use smaller plates to reduce portion sizes, create a meal schedule to avoid impulsive eating, and keep healthy snacks readily available to reduce the temptation of junk food. Recognizing emotional triggers (e.g., stress, boredom) that lead to mindless eating and replace eating with alternative activities like walking, or deep breathing are also important. In addition, they should keep unhealthy foods out of their sight, stay hydrated, keep a food diary to become more aware of what, when, and why they eat and get enough sleep as lack of sleep can disrupt hunger hormones, leading to overeating [6, 7]. 

MFE leads to permanent change in lifestyle and that is the main deference between MFE and traditional diets which aim to reduce weight in short period [2]. A study found that individuals who practiced MFE experienced a 30% reduction in emotional eating and a 25% improvement in weight control compared to those who followed traditional diet plans [8]. In an Egyptian study, about one third of the study subjects had negative attitude toward their body, and 61.4% of them reported moderate level of mindfulness of eating behavior. Positive correlation was observed between eating disorders and negative body attitude, while negative correlation was found between eating disorders and mindfulness of eating behaviors of the study subjects [9]. 

Incorporating MFE practices can be challenging with the fast-paced environments that we live in day to day. We have challenging work schedules coupled with the increase and convenience of fast-food restaurants. Living in a world with an emphasis placed on productivity increases the temptation of grabbing fast food for a quick meal or unhealthy snacking while we work [10]. The challenges of medical school pose a serious threat to the well-being of aspiring doctors [3]. Academic stress, heavy workloads, irregular schedules, the need for continuous learning, and exposure to illness and death during their education put medical students at high risk for developing mental health issues, including eating disorders [11]. The risk of eating disorders is additionally further increased by the transitional nature through young adulthood [12]. The relationship between academic stress and unhealthy eating behavior varies among individuals. While some may resort to unhealthy eating habits as a coping mechanism, others may adopt alternative strategies such as restrained eating [13–15]. 

The extent to which MFE practices are adopted may vary significantly between students in different academic disciplines. Medical students, who are exposed to extensive knowledge about health, nutrition, and the consequences of poor dietary choices, may exhibit different attitudes and behaviors toward MFE compared to their non-medical counterparts. Medical students must prioritize healthy eating habits for their well-being and the benefit of their future patients as they expected to provide effective preventive counseling to their patients as physicians. Understanding how academic discipline influences MFE behaviors can provide valuable insights into the role of education and lifestyle in shaping MFE practices, ultimately contributing to strategies that promote healthier eating behaviors among university students. So, the aim of this study was to estimate the prevalence of adoption of principles of MFE among medical and non-medical university students and to detect the effect of adoption of MFE on the body mass index (BMI) of the studied students.

### Study population and sampling technique

A cross-sectional study was conducted between February 2025 and May 2025 at Faculties of Medicine, Art, Commerce and Law, Tanta University in Egypt. Participants were selected using a non-randomized convenient sampling technique. The study criteria included: undergraduate Tanta university students of Faculties of Medicine, Art, Commerce and Law who expressed a willingness to participate.

**Table 1:** The responses of the participants to the awareness and external cues subscales of MEQ

Mindful Eating Questionnaire (MEQ)	Not-applicable %		Never/Rarely %		Sometimes %		Often %		Usually/Always %	
	Non*	M**	Non*	M**	Non*	M**	Non*	M**	Non*	M**
**Awareness**										
I notice when there are subtle flavors in the foods I eat ^#^			5.6	31.3	31.3	24.3	28.8	32.3	34.4	12.2
When eating a pleasant meal, I notice if it makes me feel relaxed ^#^			6.9	26.7	33.0	22.6	36.1	39.6	24.0	11.1
I appreciate the way my food looks on my plate ^#^			6.9	31.6	24.3	23.6	26.4	33.0	42.4	11.8
Before I eat, I take a moment to appreciate the colors and smells of my food ^#^			13.5	29.5	34.0	28.8	27.1	31.6	25.3	10.1
I notice when foods and drinks are too sweet ^#^			6.3	46.2	16.0	22.6	21.5	20.8	56.3	10.4
I taste every bite of food that I eat ^#^			27.1	14.6	28.8	36.1	21.5	27.4	22.6	21.9
I notice when the food I eat affects my emotional state ^#^			18.8	27.1	38.2	29.2	24.3	33.7	18.8	10.1
**External cues**										
At a party where there is a lot of good food, I notice when it makes me want to eat more food than I should #			37.5	19.4	31.3	28.5	21.9	31.3	9.4	20.8
I recognize when food advertisements make me want to eat #	19.8	1.4	11.1	20.8	30.2	35.1	22.2	30.9	16.7	11.8
I notice when just going into a movie theater makes me want to eat candy or popcorn #	6.6	1.4	23.6	24.3	31.9	24.7	22.6	30.9	15.3	18.8
When I eat a big meal, I notice if it makes me feel heavy or sluggish #			8.0	33.7	24.0	27.4	35.8	26.7	32.3	12.2
I recognize when I’m eating and not hungry #	18.1	5.2	14.2	14.6	36.8	33.3	19.4	26.7	11.5	20.1
I notice when I am eating from a dish of candy just because it is there #			32.3	16.7	30.6	35.1	27.1	29.9	10.1	18.4

^#^Significant difference between the two studied groups

^*^Non−medical students

^**^Medical students

Level of significance was established at *P* < 0.05

### Sample size

The formula used for the sample size calculation was: $\mathrm{n}=[Z(\sigma/\triangle)]^{2}$, where: **n** = sample size, **Z** = value representing the desired confidence level, **σ** = population standard deviation and **Δ** = precision (true value) [16]. The confidence level was set to be 95%. The Z score value for 95% confidence interval was 1.96. The precision of 5% had been decided. The standard deviation used based on the mean MFE summary score calculated among the students participated in the pilot study done before the main study (2.391 ± 0.363). Therefore, n = [1.96 (0.363/0.05)]^2^ = 202 participants + 10% drop-out added to the sample = 222 participants.

### Study tool

Data was collected using an online self- administered Google form adapted from a reliable and valid questionnaire; Mindful Eating Questionnaire (MEQ) [17, 18]. The original questionnaire was in English, translated into Arabic, and then retranslated back to English by a bilingual expert to ensure that the meaning was preserved. It was translated into Arabic to be suitable and easily filled by the non-medical students while, the English form was suitable for the medical students. The validity and reliability of the Arabic version of the questionnaire was established through a systematic process of cultural adaptation and rigorous statistical testing. Cultural nuances were addressed, and wording was adjusted to be more relevant to the non-medical Arab students. A panel of experts reviewed the preliminary Arabic version to assess its face validity (e.g., clarity and relevance) and content validity (e.g., comprehensiveness). The revised version was administered to a sample group of non-medical students to check for clarity, feasibility, and to gather data for statistical analysis. Internal consistency was measured using Cronbach’s alpha to determine if the items on the questionnaire were related and measured the same underlying concept. Test-retest reliability was measured by administering the questionnaire to the same group of students at two different times to see if the results were consistent, using the intraclass correlation coefficient (ICC). The Arabic version of the questionnaire demonstrated internal consistency (Cronbach’s alpha = 0.707) and test-retest reliability (ICC ranging from 0.657 to 0.754)]. The questionnaire form posted on students’ official and non-official social media groups. The questionnaire consisted of two sections. The first section included the socio-demographic data of the participant (age, sex, faculty, academic year, residence, self-reported body weight and height). BMI was calculated for all the participants then they were categorized according to the calculated BMI categories into two categories; < 25 (non-obese) and ≥ 25 (obese).

**Table 2:** The responses of the participants to the distraction, disinhibition and emotional response subscales of MEQ

Mindful Eating Questionnaire (MEQ)	Not-applicable %		Never/Rarely %		Sometimes %		Often %		Usually/Always %	
	Non*	M**	Non*	M**	Non*	M**	Non*	M**	Non*	M**
**Distraction**^®^										
I eat so quickly that I don’t taste what I’m eating #			5.2	37.2	18.8	18.4	27.1	33.7	49.0	10.8
My thoughts tend to wander while I am eating #			12.2	27.8	24.0	27.4	45.1	31.6	18.8	13.2
I think about things I need to do while I am eating			17.7	13.5	30.9	36.5	35.4	34.0	16.0	16.0
**Disinhibition**										
When I eat at “all you can eat” buffets, I tend to overeat ^®^^#^	32.6	6.6	33.0	33.3	31.6	29.2	0.3	0.3	2.4	30.6
When a restaurant portion is too large, I stop eating when I’m full ^#^			6.9	32.3	24.3	25.7	23.3	28.8	45.5	13.2
When I’m eating one of my favorite foods, I don’t recognize when I’ve had enough ^®^^#^			52.4	59.0	25.7	28.8	1.0	0.3	20.8	11.8
If it doesn’t cost much more, I get the larger size food or drink regardless of how hungry I feel ^®^^#^			37.5	57.3	15.3	28.1	0.7	0.0	46.5	14.6
If there are leftovers that I like, I take a second helping even though I’m full ^®^^#^			36.5	60.8	13.9	25.3	0.3	0.3	49.3	13.5
I stop eating when I’m full even when eating something I love ^**#**^			9.4	29.9	0.7	1.0	21.5	25.0	68.4	44.1
If there’s good food at a party, I’ll continue eating even after I’m full ^®^^#^			28.8	62.2	12.8	21.5	1.4	0.3	56.9	16.0
When I’m at a restaurant, I can tell when the portion I’ve been served is too large for me			15.3	15.3	1.4	0.7	28.1	27.1	55.2	56.9
**Emotional response** ^®^										
I snack without noticing that I am eating #			9.7	22.6	25.3	26.4	36.8	35.4	28.1	15.6
When I’m feeling stressed at work, I’ll go find something to eat #	33.3	0.3	19.4	34.7	26.0	28.8	13.5	17.4	7.6	18.8
When I’m sad I eat to feel better #			17.0	25.7	20.5	23.3	28.1	27.8	34.4	23.3
I have trouble not eating ice cream, cookies, or chips if they’re around the house #			10.1	26.4	17.0	25.7	21.5	33.3	51.4	14.6

^®^ Reversed before scoring (1=4, 2=3, 3=2 and 4=1)

^#^Significant difference between the two studied groups

^*^Non−medical students

^**^Medical students

Level of significance was established at *P* < 0.05

The second section included the 28-item MEQ. Its five subscales were defined as follows: ***Awareness*** subscale measuring the ability to notice the effects of food on the senses and how food affected internal states. It consisted of seven items. One of them was; “Before I eat, I take a moment to appreciate the colors and smells of my food.” ***Distraction*** subscale measuring the extent of focusing on other activities while eating. It consisted of three items. One of them was; “I think about things I need to do while I am eating.” ***Disinhibition*** subscale measuring the ability to stop eating when full. It consisted of eight items. One of them was; “If there are leftovers I like, I take a second helping even though I am full.” ***Emotional response*** subscale measuring the extent of eating in response to negative emotions. It consisted of four items. One of them was; “When I am sad, I eat to feel better.” ***External cues*** subscale measuring the extent of eating in response to environmental triggers. It consisted of six items. One of them was; “I notice when just going into a movie theater makes me want to eat candy and popcorn.”

For the MEQ, the score was calculated as previously described by **Framson** for each of the 28 items using a 4-point Likert-type scale, with the following response variants **“Never/Rarely”**,** “Sometimes”**,** “Often”** and **“Usually/Always”** [18]. Five items out of 28 had an additional response variant which was **“Not applicable”** which scored by zero. Answers to items 3, 4, 5, 8, 10, 12, 14, 15, 16, 20, 21, 22, 23, 24, 25, and 26 received scoring from 1 to 4. For items 1, 2, 6, 7, 9, 11, 13, 17, 18, 19, 27, and 28, the scoring was reversed. The maximum possible score was 4. Each subscale was calculated as the mean of the item score and the summary score was the mean of the five subscales. Questions with an inapplicable value excluded when calculated the mean of the item score. Then, the participants were divided in two categories depending on their score (cutoff point was 2). Higher scores were associated with a more MFE approach and lower scores with a less mindful approach. Therefore, if the participant’s score was more than 2, that meant he/she was adopting MFE while participant’s score equal or less than 2 meant he/she was adopting mindless eating [18]. 

### Pilot study

Pilot study was done for about 10% of the sample and its results were not included in the results of the main study. Since the questionnaire was translated into Arabic; a pilot study was conducted to assess its reliability and validity.

### Statistical analysis

Analysis was performed utilizing the Statistical Package for Social Sciences (SPSS) (version 27.0, Inc., Chicago, IL, USA). Kolmogorov-Smirnov and Shapiro-Wilk tests were used to check the normality of the quantitative data which was found to be not normally distributed; thus, quantitative data was expressed as median and inter-quartile range (IQR). Mann-Whitney U test of difference for comparison between the two studied groups was used. A descriptive analysis of all categorical variables was conducted including frequencies (n, %). The chi-square test was used to test the association between categorical outcomes. Monte-Carlo Exact test (MCET) was another test of association used when Chi-square test (X^2^) was not appropriate. Tests of correlation were used like Eta and Kendall’s tau-b to detect and describe the correlations between the different types of categorical data. Risk estimation was done using Odd’s Ratio (OR) depending on the 95% Confidence Interval (CI). Level of significance was established at *p* < 0.05.

**Table 3:** Score for Mindful Eating Questionnaire (MEQ) for the studied participants

Scores	Non-medical		Medical		Mann-Whitney U test	*P*-value
	Median	IQR^**^	Median	IQR^**^		
**Awareness**	2.785	2.428–3.142	2.285	1.857–2.571	20503.500	< 0.001*
**Distraction**	2.000	1.666–2.666	2.666	2.333–3.333	24759.500	< 0.001*
**Disinhibition**	2.875	2.571–3.250	3.125	2.750–3.375	33970.000	< 0.001*
**Emotional response**	2.250	1.750–2.666	2.750	2.250–3.000	24539.500	< 0.001*
**External cues**	2.333	2.000–2.666	2.500	2.166–2.666	37595.500	0.051
**Mindful Eating summary**	2.478	2.226–2.728	2.595	2.448–2.774	32106.000	< 0.001*

Bold values were significant *P* values

^*^ Statistically significant at P < 0.05

^**^IQR= Inter-Quartile Range

## Results

Data was collected from 288 medical students who were studying in the faculty of Medicine and 288 non-medical students who were studying in the faculties of Arts, Commerce and Law, Tanta university. Regarding the sociodemographic characteristics of the studied participants. The median age of both medical and non-medical students was 21 years (IQR = 20–21 years). Females represented nearly two thirds of the sample (63.2% of medical and 66.0% of non-medical students). Nearly half of medical students (50.7%) were from urban areas while (59.7%) of non-medical ones were from rural areas. Regarding the academic year of the participants, nearly half of the medical students (49.7%) and about two thirds of non-medical ones (60.4%) were in the third academic year.

**Table 4:** Gender comparison of score for Mindful Eating Questionnaire (MEQ)

Scores Median (IQR**)	Non-medical		*P*-value	Medical		*P*-value
	Male 98 (34.0%)	Female 190 (66.0%)		Male 106 (36.8%)	Female 182 (63.2%)	
**Awareness**	2.571 (2.142–3.035)	2.857 (2.428–3.285)	0.006*	2.142 (1.857–2.464)	2.285 (2.000–2.571.000.571)	0.211
**Distraction**	2.333 (2.000–2.666.000.666)	2.000 (1.666–2.666)	0.489	2.666 (2.250–3.333)	2.666 (2.333–3.333)	0.861
**Disinhibition**	3.000 (2.692–3.258)	2.875 (2.500–3.250)	0.117	3.125 (2.750–3.446)	3.000 (2.750–3.258)	0.025*
**Emotional response**	2.250 (1.750–2.750)	2.125 (1.750–2.666)	0.309	2.625 (2.250–3.000.250.000)	2.750 (2.250–3.000.250.000)	0.709
**External cues**	2.333 (1.958–2.666)	2.333 (2.000–2.762.000.762)	0.330	2.500 (2.166–2.666)	2.500 (2.166–2.666)	0.587
**Mindful Eating summary**	2.463 (2.252–2.734)	2.488 (2.217–2.727)	0.948	2.616 (2.420–2.775)	2.591 (2.452–2.775)	0.943

Bold values were significant *P* values for Mann−Whitney U test

^*^Statistically significant at *P* < 0.05

^**^IQR= Inter−Quartile Range

Table (1) showed the responses of both medical and non-medical students regarding the awareness and external cues subscales of MEQ. Firstly, for the awareness subscale, the table showed that, only small proportions of medical students reported **“always/usually”** used to notice when there are subtle flavors in the food they eat (12.2%), feel relaxed when they eat a pleasant meal (11.1%), appreciate the way the food looks on plate (11.8%), take a moment to appreciate the colors and smells of the food before eating (10.1%) and notice when the food affects their emotional states (10.1%) as compared to their non-medical peers who reported **“always/usually”** for those items (34.4%, 24.0%, 42.4%, 25.3% and 18.8%, respectively). Nearly half of medical students (46.1%) reported **“never/rarely”** used to notice when foods and drinks are too sweet, however more than half of non-medical ones (56.3%) reported **“always/usually”** they did. About one fifth of both medical (21.9%) and non-medical students (22.6%) reported **“always/usually”** tasting every bite of food that they eat. Secondly, for the external cues’ subscale, more than one-third of the non-medical students (37.5%) reported that they **“never/rarely”** eat more at parties because of how good the food looks as compared to the medical students who reported **“often”** and **“always/usually”** by (31.3% and 20.8%, respectively). Regarding food advertisements, the percentages of the medical students who chose **“sometimes”** and **“often”** the advertisements increase their desire to eat were (35.1% and 30.9%, respectively), while the percentages of the non-medical students who reported so were (30.2% and 22.2%, respectively). Being in environments like movie theaters **“often”** triggered the urge to eat candy or popcorn, as reported by **(**30.9%) of the medical students and **“sometimes”** triggered the urge to eat candy or popcorn, as reported by (31.9%) of the non-medical peers. Only (12.2%) of the medical students who reported **“always/usually”** response in the question about feeling heavy or sluggish after eating a large meal. In contrast, noticeable increases in **“often”** and **“always/usually”** responses were observed among the non-medical students, as reported by (35.8% and 32.3% of them, respectively). About one third of the participants (33.3% of medical students and 36.8% of non- medical students) said they **“sometimes”** used to eat although they aren’t hunger. Eating candy just because the dish is in front; was reported as **“sometimes”** by (35.1%) of the medical students, however (32.3%) of the non-medical students reported **“never/rarely”**.

Table (2) illustrated the responses of the participants regarding the distraction, disinhibition and emotional response subscales of MEQ. The table showed the extent of distraction that the participants used to have while eating; more than one third of medical students (37.2%) reported that they **“never/rarely”** eat so fast that they cannot taste the food eaten, while nearly half of non-medical students (49.0%) reported **“always/usually”** they did. About one third of medical students (31.6%) and nearly half of non-medical students (45.1%) reported that their thoughts **“often”** tend to wander while they are eating. As regards to the disinhibition subscale; nearly one third of medical students reported **“always/usually”** for tending to overeat when eating at “all you can eat” buffets (30.6%), while only (2.4%) of non-medical students reported so. **“Never/rarely”** was reported by medical students as regards stopping eating if they are full when a restaurant portion is too large (32.3%) and when eating something they love (29.9%) while non-medical ones reported **“always/usually”** (45.5% and 68.4%, respectively). Medical students (20.8%) reported **“always/usually”** for not recognizing when they have had enough when eating their favorite food, while (11.8%) of non-medical students reported so. The percentage of medical students reported **“never/rarely”** for getting larger size food or drink regardless of how hungry they feel if it doesn’t cost much more was (57.3%), while non-medical reported **“always/usually”** (46.5%). Nearly two thirds of medical students (60.8%) reported **“never/rarely”** taking a second helping even though being full if there are leftovers that they like and nearly half of non-medical ones (49.3%) reported **“always/usually”**. Also, nearly two thirds of medical students (62.2%) reported **“never/rarely”** for continuing eating even if they are full when there is a good food at a party and more than half of non-medical ones (56.9%) reported **“always/usually”**. Regarding the emotional response subscale, more than one third of both medical and non-medical students (35.4% and 36.8%, respectively) reported **“often”** snacking without noticing that they are eating. In response to stress at work; (34.7%) of medical students and (19.4%) of non-medical students reported **“never/rarely”** go find something to eat. Feeling sad was **“often”** a trigger for (27.8%) of medical students to eat in order to feel better while it was **“always/usually”** a trigger for (34.4%) of non-medical students to eat in order to feel better. One third of medical students (33.3%) reported **“often”** having trouble not eating ice cream, cookies or chips if they are around the house while, (51.4%) of non-medical students reported that they **“always/usually”** having the same trouble.

**Table 5:** Relation between socio-demographic characteristics of the participants and Mindful Eating summary score

**Characteristic**				**Mindful eating summary score**		**X**^2^	**P-value**	**OR**	**95% CI**
				**N** **(%)**					
		**Total ** **(576)**		**Mindless eating {≤ 2}** **32** **(5.6)**	**Mindful eating {>2}** **544** **(94.4)**				
		**n**	**%**						
**Sex **	Male	**204**	**35.4**	10 (4.9)	194 (95.1)	0.257	0.612	0.820	0.381–1.767.381.767
	Female	**372**	**64.6**	22 (5.9)	350 (94.1)				
**Residence **	Rural	**314**	**54.5**	16 (5.1)	298 (94.9)	0.278	0.598	0.826	0.405–1.684.405.684
	Urban	**262**	**45.5**	16 (6.1)	246 (93.9)				
**Faculty**	Non-Medical	**288**	**50.0**	25 (8.7)	263 (91.3)	10.721	0.001*	**3.816**	**1.623–8.971.623.971**
	Medical	**288**	**50.0**	7 (2.4)	281 (97.6)				
**Academic year**	1^st^	**64**	**11.1**	1 (1.6)	63 (98.4)	6.611**	0.158		
	2^nd^	**74**	**12.8**	6 (8.1)	68 (91.9)				
	3^rd^	**317**	**55.1**	22 (6.9)	295 (93.1)				
	4^th^	**94**	**16.3**	3 (3.2)	91 (96.8)				
	5^th^	**27**	**4.7**	0 (0.0)	27 (100.0)				

Bold values were significant *P *value

^*^Statistically significant at P < 0.05

^**^MCET = Monte Carlo Exact test

Table (3) illustrated the medians and IQRs of the summary score of MEQ and its five subscales for studied medical and non-medical participants. Medians of the MFE summary score and four subscales of MEQ [Distraction, Disinhibition, Emotional response & External cues] were higher for medical students than their non-medical peers. On the other hand, the median of the awareness score was higher for non-medical students than its median for medical students. There were significant differences between the medical and non-medical studied participants as regards the summary score and four subscales of MEQ [Awareness, Distraction, Disinhibition & Emotional response]; however, no statistically significant difference was detected between the two groups as regards the External cues subscale.

Table (4) illustrated the medians and IQRs of the summary score of MEQ and its five subscales for studied medical and non-medical participants taking in consideration the sex distribution among the studied groups. The sex distribution of the study participants was fairly balanced between the two studied groups, showing no statistically significant difference (X^2^ = 0.486, P-value = 0.486) with a female predominance representing (66.0%) of non-medical studied subjects and (63.2%) of medical studied subjects. Regarding the non-medical studied participants, there was a statistically significant difference between the males and females for the awareness subscale of MEQ (P-value = 0.006) while no statistically significant differences were detected between them as regards the MFE summary score and the four subscales of MEQ [Distraction, Disinhibition & Emotional response & External cues]. Non-medical females showed higher scores for the awareness subscale than males. For the medical studied participants, there was a statistically significant difference between the two sexes as regards the disinhibition subscale of MEQ (P-value = 0.025) while no statistically significant differences were detected between the them as regards the MFE summary score and the four subscales of MEQ [Awareness, Distraction, Emotional response & External cues]. Medical males showed higher scores for the disinhibition subscale than females.

Table (5) showed that the majority of the studied subjects (94.4%) were used to adopt MFE. It showed that (95.1%) of the participated male students were adopting MFE and (94.1%) of the participated female students were also adopting it. No statistically significant difference was detected between the sex of the participants and their MFE summary score categories (P-value = 0.612). Regarding the residence of the participants, most of the students whether living in rural areas (94.9%) or in urban areas (93.9%) were used to adopt MFE. No statistically significant difference was detected between the residence of the participants and their MFE summary score categories (P-value = 0.598). The percentages of medical and non-medical students were used to adopt MFE were (97.6% and 91.3%, respectively). The association between the type of the study (faculty) and adoption of MFE was statistically significant (P-value = 0.001). The effect of the type of study (faculty) of the participants on their MFE summary score categories was small (Squared Eta = 0.018). The non-medical study makes the student more prone for adopting mindless eating by 3.8 folds more than the medical study. Therefore, medical study permits adopting MFE by 73.8% more than non-medical studies. The majority of the students of the third academic year (93.1%) who representing more than half of the participants (55.1%) were used to adopt MFE, however no statistically significant difference was detected between the academic year and the MFE summary score categories (P-value = 0.158).

Table (6) showed that nearly three fourths of the studied subjects (74.3%) had BMI ≥ 25. It showed that (72.5%) of the participated male students and (75.3%) of the participated female students had BMI ≥ 25. No statistically significant difference was detected between the sex of the participants and their BMI categories (P-value = 0.510). Regarding the residence of the participants, most of the students living in rural areas (78.3%) had BMI < 25 as compared to their peers who were living in urban areas where nearly one third of them (30.5%) had BMI ≥ 25. The association between the residence of the participants and BMI categories was statistically significant (P-value = 0.015). The effect of the residence of the participants on their BMI categories was small (Squared Eta = 0.010). The percentage of medical students (17.4%) having BMI ≥ 25 was less than the percentage of their non-medical peers (34.0%). The association between the type of the study (faculty) and BMI was statistically significant (P-value = < 0.001). The effect of the type of study (faculty) of the participants on their BMI categories was moderate (Squared Eta = 0.036). The non-medical study makes the student more prone for obesity by 0.6-fold more than the medical study. Therefore, medical study protects from obesity by about 60% more than non-medical studies. The majority of the students of the third academic year (70.7%) who representing more than half of the participants (55.1%) had BMI ≥ 25 and the association between the academic year of the participants and their BMI categories was statistically significant (P-value = 0.010). A significant positive weak correlation was detected between the academic year of the participants and BMI categories (value of Kendall’s tau-b was 0.100, P-value = 0.005). The table showed that higher scores on the MFQ overall and on each of the categories except the awareness score had been associated with lower BMI. Most of the students adopting MFE (75.4%) had BMI < 25 as compared to their peers who did not adopt MFE where more than one third of them (43.8%) had BMI ≥ 25. The association between MFE summary score categories and BMI categories was statistically significant (P-value = 0.016). Furthermore, a significant negative weak correlation was detected between MFE summary score categories and BMI categories (value of Kendall’s tau-b was – 0.100, P-value = 0.044). Thus, adoption of MFE protecting from being obese by 58% suggesting that MFE may play an important role in long-term weight maintenance.

**Table 6:** The effect of adoption of mindful eating on the body mass index of the studied students

**Characteristic**				**Body Mass Index (BMI)**		**X**^2^	**P-value**	**OR**	**95% CI**
				**N** **(%)**					
		**Total ** **(576)**		**Non- obese** **< 25** **428** **(74.3)**	**Obese** **≥ 25** **148** **(25.7)**				
		**N**	**%**						
**Sex **	Male	**204**	**35.4**	148 (72.5)	56 (27.5)	0.510	0.475	0.868	0.590–1.279.590.279
	Female	**372**	**64.6**	280 (75.3)	92 (24.7)				
**Residence **	Rural	**314**	**54.5**	246 (78.3)	68 (21.7)	5.897	0.015*	**1.590**	**1.092–2.316.092.316**
	Urban	**262**	**45.5**	182 (69.5)	80 (30.5)				
**Faculty**	Non-medical	**288**	**50.0**	190 (66.0)	98 (34.0)	20.951	<0.001*	**0.407**	**0.276–0.602.276.602**
	Medical	**288**	**50.0**	238 (82.6)	50 (17.4)				
**Academic year**	1^st^	**64**	**11.1**	58 (90.6)	6 (9.4)	13.267	0.010*		
	2^nd^	**74**	**12.8**	59 (79.7)	15 (20.3)				
	3^rd^	**317**	**55.1**	224 (70.7)	93 (29.3)				
	4^th^	**94**	**16.3**	66 (70.2)	28 (29.8)				
	5^th^	**27**	**4.7**	21 (77.8)	6 (22.2)				
**Awareness score**	Mindless eating {≤ 2}	**133**	**23.1**	109 (82.0)	24 (18.0)	5.300	0.021*	**1.765**	**1.084–2.876.084.876**
	Mindful eating {>2}	**443**	**76.9**	319 (72.0)	124 (28.0)				
**Distraction score**	Mindless eating {≤ 2}	**217**	**37.7**	151 (69.6)	66 (30.4)	4.063	0.044*	**0.677**	**0.463–0.990.463.990**
	Mindful eating {>2}	**359**	**62.3**	277 (77.2)	82 (22.8)				
**Disinhibition score**	Mindless eating {≤ 2}	**22**	**3.8**	12 (54.5)	10 (45.5)	4.678	0.031*	**0.398**	**0.168–0.942.168.942**
	Mindful eating {>2}	**554**	**96.2**	416 (75.1)	138 (24.9)				
**Emotional response score**	Mindless eating {≤ 2}	**181**	**31.4**	122 (67.4)	59 (32.6)	6.586	0.010*	**0.601**	**0.407–0.889.407.889**
	Mindful eating {>2}	**395**	**68.6**	306 (77.5)	89 (22.5)				
**External cues score**	Mindless eating {≤ 2}	**141**	**24.5**	104 (73.8)	37 (26.2)	0.029	0.864	0.963	0.625–1.484.625.484
	Mindful eating {>2}	**435**	**75.5**	324 (74.5)	111 (25.5)				
**Mindful eating summary score**	Mindless eating {≤ 2}	**32**	**5.6**	18 (56.3)	14 (43.8)	5.785	0.016*	**0.420**	**0.203–0.868.203.868**
	Mindful eating {>2}	**544**	**96.4**	410 (75.4)	134 (24.6)				

Bold values were significant *P *values

^*^Statistically significant at *P *< 0.05

## Discussion

- Adoption of principles of MFE among medical and non-medical students:

The majority of the studied subjects were used to adopt MFE. In our study the median of MFE summary score among the medical students was 2.595 (2.448–2.774) and among the non-medical students was 2.478 (2.226–2.728). A study done among Romanian medical students, a mean mindfulness level of 2.8 ± 0.3 was detected [19]. Another research conducted by Moore et al., (2013) reported a mean mindfulness level of 2.89 ± 0.32 among medical students [20]. The association between the type of the study (faculty) and adoption of MFE was statistically significant among the participants of our research. Also, the medians of the four subscales of MEQ [Distraction, Disinhibition, Emotional response & External cues] were higher for medical students than their non-medical peers. On the other hand, the median of the awareness score was higher for non-medical students than its median for medical students. That discrepancy might be explained by; some of the participants might give inaccurate or socially desirable responses giving what is called “respondent bias”. Respondent bias is considered one of the disadvantages of questionnaires. That explanation was confirmed as in our study; the higher scores on the MFQ overall and on each of the categories except the awareness score had been associated with lower BMI. In a study done among Romanian medical students; an increase in the nutrition knowledge score corresponded to an increase in the mindful eating score. However, the clinical significance of this association may be limited due to the study’s design [19]. Aggarwal et al. (2020) found that medical doctors generally have lower levels of nutrition knowledge, particularly regarding expert recommendations [21]. 

There is a scarcity of the literature examining the connection between nutrition knowledge and mindful eating. In research conducted by Moore et al., (2013), a 1 SD increase in the nutrition knowledge score corresponded to a 0.240 SD increase in the mindful eating score [20]. Kurtipek et al. (2020) conducted a cross-sectional study investigating this association and found that undergraduate sports students exhibited a shared variance level of less than 1% [22]. Romanian medical students identified a higher level of shared variance (4%) [19]. 

Considering sex differences, the medical male participants, showed higher scores for the disinhibition subscale than medical females. Males usually eat in a practical manner, eating only when they are hungry and stop eating when full. This behavior is obviously evident among medicals. However, females prefer eating at “all you can eat” buffets, gatherings and parties. Females also might be concerned more by the financial aspect. It is no bother for females to eat leftovers. Furthermore, non-medical female participants, showed higher scores for the awareness subscale than non-medical males. Females usually eat for psychological relief and relaxation. They might have more time compared to males to appreciate the way their food looks on the plate. Females are responsible for processing, preparing food and dealing much with food so, they could take their time appreciating the colors and smells of their food, tasting every bite of food that they eat.

- Effect of adoption of MFE on the BMI of the studied students:

Higher scores on the MFQ overall and on each of the categories except the awareness score had been associated with lower BMI. Similar to our findings, previous research has established a negative and significant relationship between MFE and BMI. Cross-sectional studies conducted with medical students [20] and the general population [17, 23] have consistently shown that higher BMI correlates with lower levels of mindful eating. Among the Romanian medical students, mindless eating emerged as a predictor of excess weight with each 1-point decrease in the mindful eating score associated with 7.6 times increase in the likelihood of having excess weight [19]. Intervention studies have further demonstrated that increasing mindfulness can lead to weight loss [24]. Studies investigating the association between nutrition knowledge and obesity have yielded mixed results, potentially influenced by sampling and sociodemographic factors. While some studies reported nonsignificant associations [25, 26], others found significant links [27, 28]. Longitudinal studies have indicated that higher nutrition knowledge may lead to reduced BMI levels [29–31]., yet the mindful aspects of eating have not been extensively addressed in these studies. In a multivariate model, high weight gain remains independent of mindful eating. However, univariate testing reveals a significant association with disinhibition and emotional response [32]. Reviews and meta-analyses examining the effects of mindfulness interventions on individuals with excess weight acknowledge the roles of disinhibition and emotional eating in weight gain over time [33, 34]. 

A significant positive weak correlation was detected between the academic year of the participants and BMI categories. Our findings agreed with the findings detected among the Romanian students indicating that students in clinical years are 2.2 times more likely to report excessive weight compared to their nonclinical peers; a trend consistent with the findings of Leventer-Roberts et al. (2021), where third-year medical residents were 2.2 times more likely to be overweight than first-year residents [35]. The demanding nature of medical training poses challenges in maintaining healthy habits such as balanced nutrition, adequate sleep, and regular physical activity, all of which are associated with weight gain [21] and burnout [36]. 

### Limitations

The study was conducted at a single university, which may limit the generalizability of the findings to other populations or regions. Respondent bias may be present, as some of the participants may give inaccurate or socially desirable responses causing skewed results. Additionally, a selection bias may be present, as students with an increased interest in nutrition and healthy living might be more inclined to participate. Furthermore, relying on self-reported weight and height poses a limitation to the study’s accuracy.

### Future research

Further studies are needed to assess the nature of the relation between the awareness, the external cues subscales and type of the study (faculty) of the university students and their BMI.

### Recommendations

Orientation campaigns on principles of MFE is needed to encourage healthier food choices and portion control. This orientation can be provided through elective courses; we can add a curriculum to non-medical colleges, cross lectures between colleges, and conferences. Organization of interactive sessions is recommended to help students recognize and manage emotional and social triggers that lead to mindless eating. Addressing work-related lifestyle factors contributing to weight gain among medical professionals is indicated, such as irregular meal schedules and high-stress levels. According to the addressed factors, consider modifications in the curriculum to ensure a balanced workload, preventing excessive stress and burnout among students. In addition, incorporate structured stress management and resilience training modules within the curriculum to equip students with effective coping strategies for high-pressure environments. Therefore, integrating mindfulness principles into interventions can yield positive outcomes, such as promoting MFE behaviors and contributing to weight control. This may cause enhancements in emotional eating, binge eating, and satiety cues while fostering a healthier relationship with food and body image.

## Conclusion

Notable disparities were revealed in MFE summary score and four key subscales of MEQ; awareness, distraction, disinhibition, emotional response whereas the external cues subscale showed no significant variation between the medical and non-medical students. The study underscored a potential link between MFE and lower BMI, suggesting that adopting MFE practices may contribute to better weight management. Students who practiced MFE were less likely to fall into the obese category compared to those who did not. Such findings suggested that academic background played a substantial role in shaping dietary behaviors and reinforced the potential of MFE as a viable strategy for promoting healthier eating habits and effective weight management among university students.

## Data Availability

All data generated or analysed during this study are included in this published article and its supplementary information files.

## Abbreviations

BMI: Body Mass Index
CI: Confidence Interval
IQR: Inter-Quartile Range
MCET: Monte-Carlo Exact test
MFE: Mindful eating
MEQ: Mindful Eating Questionnaire
OR: Odd’s Ratio
SPSS: Statistical Package for Social Sciences
